# Comparative Effectiveness of Opioid Tapering or Abrupt Discontinuation vs No Dosage Change for Opioid Overdose or Suicide for Patients Receiving Stable Long-term Opioid Therapy

**DOI:** 10.1001/jamanetworkopen.2022.26523

**Published:** 2022-08-12

**Authors:** Marc R. Larochelle, Sara Lodi, Shapei Yan, Barbara A. Clothier, Elizabeth S. Goldsmith, Amy S. B. Bohnert

**Affiliations:** 1 Clinical Addiction Research and Education Unit, Section of General Internal Medicine, Boston University School of Medicine, Boston Medical Center, Boston, Massachusetts; 2 Department of Biostatistics, Boston University School of Public Health, Boston, Massachusetts; 3 Center for Care Delivery and Outcomes Research, Minneapolis Veterans Affairs Health Care System, Minneapolis, Minnesota; 4 Minneapolis Veterans Affairs Health Care System, Minneapolis, Minnesota; 5 Department of Medicine, University of Minnesota Medical School, Minneapolis; 6 Department of Anesthesiology, University of Michigan, Veterans Affairs Center for Clinical Management Research, Ann Arbor

## Abstract

**Importance:**

Opioid dosage tapering has emerged as a strategy to reduce harms associated with long-term opioid therapy; however, evidence supporting this approach is limited.

**Objective:**

To identify the association of opioid tapering or abrupt discontinuation with opioid overdose and suicide events among patients receiving stable long-term opioid therapy without evidence of opioid misuse.

**Design, Setting, and Participants:**

This comparative effectiveness study with a trial emulation approach used a large US claims data set of individuals with commercial insurance or Medicare Advantage who were aged 18 years or older and receiving stable long-term opioid therapy without evidence of opioid misuse between January 1, 2010, and December 31, 2018. Statistical analysis was performed from January 17, 2020, through November 12, 2021.

**Interventions:**

Three opioid dosage strategies: stable dosage, tapering (dosage reduction ≥15%), or abrupt discontinuation.

**Main Outcomes and Measures:**

Time to opioid overdose or suicide event identified from *International Classification of Diseases, Ninth Revision* and *International Statistical Classification of Diseases and Related Health Problems, Tenth Revision* diagnosis codes in medical claims over 11 months of follow-up. Inverse probability weighting was used to adjust for baseline confounders. The primary analysis used an intention-to-treat approach; follow-up after assignment regardless of changes in opioid dose was included. A per-protocol analysis was also conducted, in which episodes were censored for lack of adherence to assigned treatment.

**Results:**

A cohort of 199 836 individuals (45.1% men; mean [SD] age, 56.9 [12.4] years; and 57.6% aged 45-64 years) had 415 123 qualifying, long-term opioid therapy episodes; 87.1% of episodes were considered stable, 11.1% were considered a taper, and 1.8% were considered abrupt discontinuation. The adjusted cumulative incidence of opioid overdose or suicide events 11 months after baseline was 0.96% (95% CI, 0.92%-0.99%) with a stable dosage strategy, 1.10% (95% CI, 0.99%-1.22%) with a tapered dosage strategy, and 1.28% (95% CI, 0.93%-1.38%) with an abrupt discontinuation strategy. The risk difference between a taper and a stable dosage was 0.15% (95% CI, 0.03%-0.26%), and the risk difference between abrupt discontinuation and a stable dosage was 0.33% (95% CI, −0.03% to 0.74%). Results were similar using the per-protocol approach.

**Conclusions and Relevance:**

This study identified a small absolute increase in risk of harms associated with opioid tapering compared with a stable opioid dosage. These results do not suggest that policies of mandatory dosage tapering for individuals receiving a stable long-term opioid dosage without evidence of opioid misuse will reduce short-term harm via suicide and overdose.

## Key Points

**Question:**

For patients receiving stable long-term opioid therapy compared with a stable opioid dosage, what is the association of opioid dosage tapering or abrupt discontinuation with opioid overdose or suicide?

**Findings:**

In this comparative effectiveness study of 415 123 episodes of stable long-term opioid therapy among 199 836 individuals, opioid tapering was associated with a small absolute increase in opioid overdose or suicide compared with a stable opioid dosage. No significant difference in outcomes between abrupt discontinuation and stable opioid therapy was identified.

**Meaning:**

These findings do not support opioid dosage tapering as a strategy to reduce harms for patients receiving stable long-term opioid therapy without evidence of misuse.

## Teaser

This comparative effectiveness study examines the association of opioid tapering or abrupt discontinuation with opioid overdose and suicide events among patients receiving a stable long-term opioid dosage without evidence of opioid misuse.

## Introduction

Evidence to support the benefits of opioid therapy for chronic pain is lacking, and opioid analgesics are associated with increased risk of opioid-related harms, including opioid overdose death.^1,2,3^ The 2016 Centers for Disease Control and Prevention (CDC) Guideline for Prescribing Opioids for Chronic Pain recommended tapering opioid dosages if benefits no longer outweigh harms.^4^ In response, some health systems and US states enacted stringent dose limits that were applied with few exceptions regardless of individual patients’ risk of harms.^5,6,7^ These efforts were associated with declines in opioid prescribing and dosages in the United States.^8,9,10^

A systematic review of opioid tapering studies found low-quality evidence favoring tapering in improving pain intensity, function, and quality of life.^11^ Meanwhile, increasing reports of patients experiencing adverse effects from forced opioid tapers, including opioid withdrawal, increasing pain, decreasing function, and suicidality, has led pain experts, authors of the CDC guideline, and the US Food and Drug Administration to issue warnings of the harms of rapid opioid tapers.^12,13,14^

Several observational studies have identified harms associated with opioid tapering and discontinuation, but they also have several limitations.^15,16,17,18^ Many studies focused on opioid discontinuation, which is likely more destabilizing than gradual tapering. Furthermore, there is a high potential for confounding. For example, clinicians may be more likely to select tapering for individuals who have developed an opioid use disorder (OUD) or opioid misuse, and these individuals have greater risk of suicide and overdose.^19,20,21^ For individuals with evidence of opioid misuse, guidelines recommend intervention beyond tapering, including evaluation for and treatment of OUD along with transition to a medication for OUD.^4^ We sought to overcome these limitations using data from a large claims database to compare the association of opioid tapering, abrupt discontinuation, or stable opioid therapy with opioid overdose or suicidal ideation or attempt among patients receiving stable long-term opioid dosages without signs of OUD or opioid misuse. To strengthen our inference, we used the target trial approach to study design and statistical analysis of observational data.^22^

## Methods

### Study Design and Data Source

We conducted a comparative effectiveness study using the deidentified Optum Clinformatics Data Mart Database that includes adjudicated pharmacy, outpatient, and inpatient medical claims for individuals with commercial or Medicare Advantage insurance in all 50 states, the District of Columbia, and Puerto Rico. We included data from January 1, 2010, through December 31, 2018. We adapted the emulated trial protocol proposed to study opioid tapering published by the National Academies of Sciences, Engineering, and Medicine in 2019.^23^ The University of Michigan institutional review board determined this study to be exempt owing to lack of involvement of human participants; patient consent was waived because the data were deidentified. This study followed the International Society for Pharmacoeconomics and Outcomes Research (ISPOR) reporting guideline for comparative effectiveness research.^24^

### Eligibility Criteria

**Figure 1.**
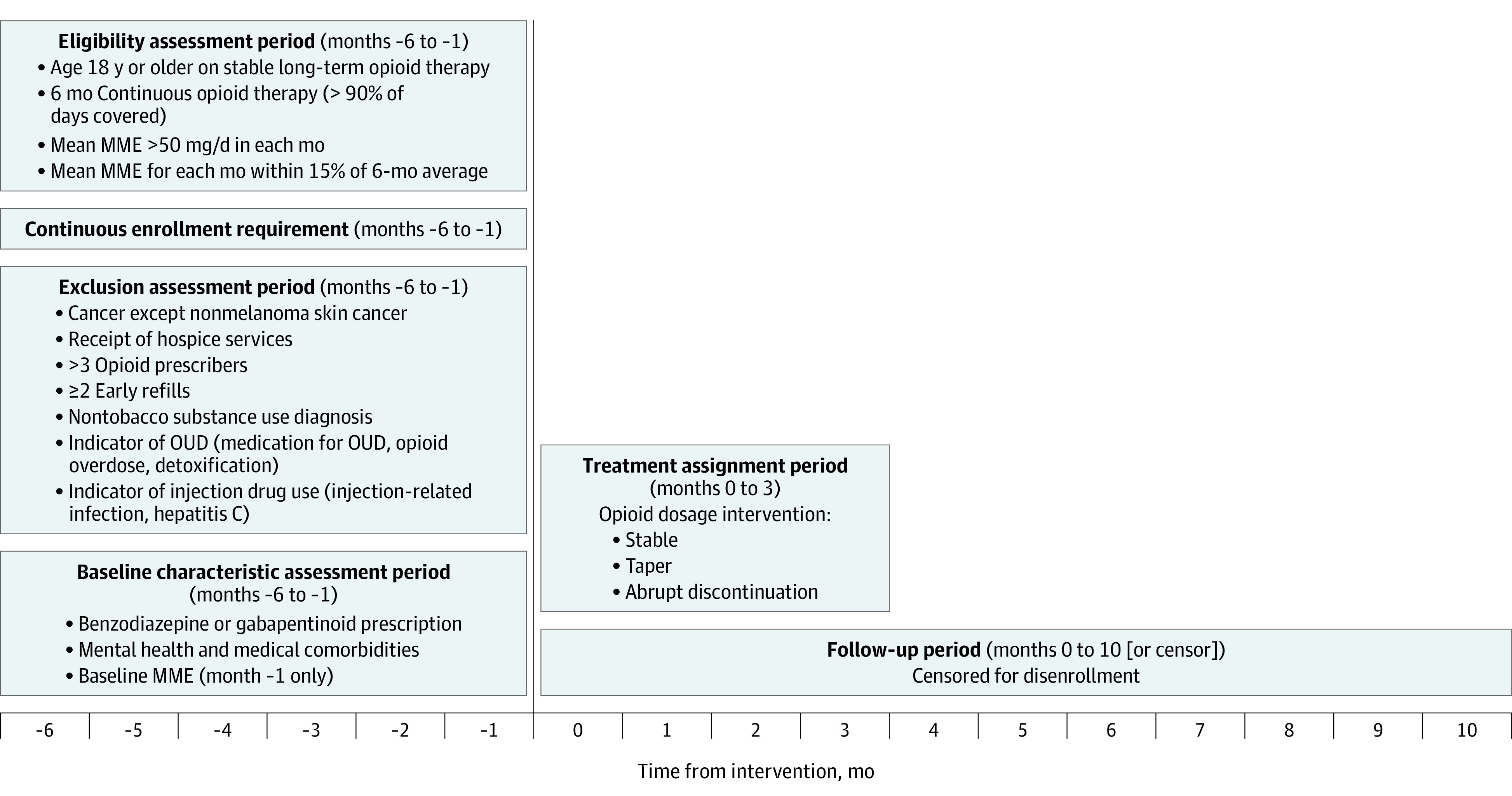
Graphical Depiction of Study Design, Including Time Periods for Identifying Study Eligibility, Exclusion Criteria, Baseline Characteristics, Treatment Assignment, and Outcome Follow-up MME indicates morphine milligram equivalent; OUD, opioid use disorder.

We adapted a previously used approach to identify stable long-term opioid therapy.^8^ First, using opioid prescription pharmacy claims, we calculated the mean daily morphine milligram equivalent (MME) for all enrollees during the 9-year study period (eAppendix in the Supplement). Stable long-term opioid therapy was defined using the following criteria during a 6-month eligibility assessment period: (1) continuous opioid therapy, defined as having 90% of days or more with a mean MME of more than 0; (2) mean MME for each month of 50 mg or more per day; and (3) mean MME for each month varied by no more than 15% above or below the 6-month mean. We excluded individuals younger than 18 years and those without continuous enrollment for the eligibility assessment period plus 1 month of follow-up (Figure 1).

Exclusion criteria were selected to focus on patients for whom tapering or discontinuation of opioids would be clinically appropriate options and applied during the 6-month eligibility assessment period. We excluded individuals with cancer except nonmelanoma skin cancer or receipt of hospice services (eTables 1 and 2 in the Supplement). We excluded individuals with evidence that they may already be experiencing opioid-related harms, as the intervention should also include evaluation for and treatment of OUD. We excluded individuals with more than 3 prescribers or 2 or more early refills, which are opioid dispensing patterns associated with opioid overdose (eAppendix in the Supplement).^25,26^ We excluded individuals with a diagnosis of substance use, abuse, or dependence, indicators that are potentially consistent with OUD or injection drug use, including receipt of medication for OUD, opioid overdose, detoxification, or an injection-related infection, including hepatitis C (eTables 1 and 2 in the Supplement).

We included multiple episodes of stable long-term opioid therapy for individuals who met the eligibility criteria more than once. We included only the first qualifying episode in each year and excluded episodes with overlap in the 6-month eligibility assessment period.

### Outcome and Follow-up

The primary outcome was a composite event, encompassing a medical claim for opioid overdose or a suicide event (suicidal ideation or attempt) identified using *International Classification of Diseases, Ninth Revision* and *International Statistical Classification of Diseases and Related Health Problems, Tenth Revision* diagnosis codes (eTable 1 in the Supplement).^27^ Follow-up started after baseline, the last month of a qualifying eligibility assessment period, and ended at the earliest month of outcome, death, loss to follow-up due to to health plan disenrollment, or end of follow-up (month 10; Figure 1).

### Treatment Strategies and Assignment

We compared 3 treatment strategies during a 4-month treatment strategy assignment period (ie, grace period) after baseline: (1) tapering, (2) abrupt discontinuation, and (3) stable dosage (Figure 1). We defined tapering as 2 consecutive months with a mean MME reduction of 15% or more compared with the baseline month. We required 2 consecutive months of dosage change to classify tapering after reviewing dosage trajectories; a single month with a 15% dosage reduction frequently appeared to occur because of a delay in filling a prescription rather than an attempt to taper. We classified episodes meeting tapering criteria as abrupt discontinuation if the second qualifying month had an MME equal to 0 mg. We defined stable dosage as no taper or abrupt discontinuation.

Because we used a 4-month grace period to assign treatment strategies and outcome events occurred during this grace period, we used cloning to avoid immortal time and other potential biases.^22,28,29,30^ More specifically, we created 3 clones of each episode, with each clone assigned to 1 of the 3 treatment strategies. We censored clones when the opioid dosage trajectory was no longer consistent with the assigned strategy, with a single clone remaining at the end of the 4-month grace period. Illustrative examples of dosage trajectories for treatment assignment and clone censoring are included in the eFigure in the Supplement.

### Causal Contrast

We estimated the equivalent of the intention-to-treat effect of the target trial; once an episode became associated with a single treatment strategy, it remained assigned to that treatment strategy for the remainder of follow-up. As a secondary analysis, we estimated the equivalent of the per-protocol effect of the target trial by further adjusting our estimates for factors associated with lack of compliance with the assigned treatment strategy. More specifically, we censored follow-up as stated: (1) episodes assigned to a stable dosage were censored if the opioid dosage dropped 15% below the baseline mean for 2 consecutive months, (2) episodes assigned to a taper strategy were censored if the dosage returned to within 15% of the final baseline month, and (3) episodes assigned to abrupt discontinuation were censored if the MME was greater than 0 in any month (eFigure in the Supplement).

### Potential Confounders

We adjusted our estimates for demographic and treatment characteristics evaluated during the eligibility assessment period: baseline year dichotomized as 2010 to 2015 vs 2016 to 2018 to account for conversion from *International Classification of Diseases, Ninth Revision* to *International Statistical Classification of Diseases and Related Health Problems, Tenth Revision* in October 2015 and release of the CDC guidelines on opioid tapering in early 2016; Census region; insurance plan type (commercial vs Medicare Advantage); age; sex; race and ethnicity to capture unmeasured social factors, including the experience of racism; MME range in the baseline month (50-89 mg, 90-199 mg, or ≥200 mg); benzodiazepine prescription; gabapentin or pregabalin prescription (eTable 3 in the Supplement); depression, anxiety, attention-deficit/hyperactivity disorder, posttraumatic stress disorder, bipolar disorder, and psychosis; and a modified Elixhauser comorbidity score for medical comorbid conditions, excluding psychiatric diagnoses included separately (eTable 4 in the Supplement).^31^

### Statistical Analysis

Statistical analysis was performed from January 17, 2020, through November 12, 2021. We present descriptive statistics for the overall cohort and by treatment strategy at the end of the treatment assignment period. We present unadjusted dosage trajectories for each clone through month 9 of follow-up for the intention-to-treat and per-protocol approaches separately.

We used methods for survival analysis and used inverse probability weighting to adjust for potential confounding. We calculated 3 sets of time-varying weights: treatment weights to adjust for censoring of clones that did not adhere to their assigned treatment strategy, censoring weights to adjust for selection bias from loss to follow-up, and treatment adherence weights used only in the per-protocol analysis. Each weight was independently calculated as the inverse probability of not being censored using regression models as specified in the eAppendix in the Supplement and a single summary weight for each month and clone by multiplying the weights together.

For each outcome, we fit a weighted pooled logistic regression model with an inverse of probability weight, indicator variables for treatment strategy, month, and an interaction term between month and treatment strategy. We used the model’s predicted probabilities to estimate the cumulative incidence of each outcome 10 months after baseline for each treatment strategy. We also calculated the risk difference and risk ratio to compare taper and abrupt discontinuation vs stable treatment strategies at month 10. We used a nonparametric bootstrap procedure based on 500 samples to obtain percentile-based 95% CIs. We used 2-tailed tests with a significance level of .05. All analyses were conducted with SAS, version 9.4 (SAS Institute Inc).

### Secondary and Sensitivity Analyses

We repeated the intention-to-treat approach for overdose and suicide outcomes separately. We conducted stratified analyses by baseline MME and year to assess for effect modification. We also conducted 2 sensitivity analyses. First, we trimmed weights at 99% to assess the association of extreme outliers with model estimates. Second, we used a less restrictive approach to treatment assignment, requiring only a single month of dosage reduction to qualify as a taper or abrupt discontinuation.

## Results

We identified 260 508 individuals with 599 999 episodes of stable long-term opioid therapy. After applying exclusion criteria, the cohort consisted of 199 836 individuals (45.1% men; mean [SD] age, 56.9 [12.4] years; and 57.6% aged 45-64 years) with 415 123 qualifying long-term opioid therapy episodes (Table 1). Baseline MME was 50 to 89 mg per day for 41.2% of the cohort, 90 to 199 mg per day for 35.4%, and 200 or more mg per day for 23.5%. A total of 34.8% of individuals received benzodiazepine prescriptions, 18.0% had a comorbid diagnosis of anxiety, and 19.7% had a comorbid diagnosis of depression during the 6-month eligibility assessment period.

**Table 1.** Baseline Characteristics for the Full Cohort and by Treatment Group in the First Month After the Treatment Assignment Period

Baseline characteristic	Patients, No. (%)				
	Full cohort		Treatment group^a^		
	Patients (N = 199 836)^b^	Eligible episodes (N = 415 123)^b^	Stable dosage (n = 332 121)	Taper (n = 42 246)	Abrupt discontinuation (n = 6886)
Male	90 177 (45.1)	189 491 (45.6)	152 330 (45.9)	18 052 (42.7)	3347 (48.6)
Age, y					
18-44	31 713 (15.9)	54 935 (13.2)	41 093 (12.4)	5588 (13.2)	1105 (16.0)
45-64	115 198 (57.6)	244 651 (58.9)	196 934 (59.3)	24 068 (57.0)	3628 (52.7)
≥65	52 924 (26.5)	115 537 (27.8)	94 094 (28.3)	12 590 (29.8)	2153 (31.3)
Race and ethnicity					
Asian	1546 (0.8)	3179 (0.8)	2518 (0.8)	358 (0.8)	41 (0.6)
Non-Hispanic					
Black	21 675 (10.8)	43 606 (10.5)	34 739 (10.5)	4771 (11.3)	744 (10.8)
White	142 671 (71.4)	302 614 (72.9)	242 191 (72.9)	30 377 (71.9)	4922 (71.5)
Hispanic	12 304 (6.2)	25 541 (6.2)	20 624 (6.2)	2587 (6.1)	386 (5.6)
Unknown	21 639 (10.8)	40 183 (9.7)	32 049 (9.6)	4153 (9.8)	793 (11.5)
Baseline MME, mg^c^					
50-89	82 274 (41.2)	154 345 (37.2)	125 170 (37.7)	13 670 (32.4)	2973 (43.2)
90-199	70 660 (35.4)	153 308 (36.9)	122 220 (36.8)	16 065 (38.0)	2503 (36.3)
≥200	46 901 (23.5)	107 470 (25.9)	84 731 (25.5)	12 511 (29.6)	1410 (20.5)
Benzodiazepine prescription^d^	69 470 (34.8)	149 020 (35.9)	117 597 (35.4)	15 832 (37.5)	2498 (36.3)
Gabapentinoid prescription^d^	64 139 (32.1)	132 899 (32.0)	105 798 (31.9)	14 309 (33.9)	2242 (32.6)
Depression^e^	39 280 (19.7)	79 925 (19.3)	62 967 (19.0)	8935 (21.1)	1392 (20.2)
Anxiety^e^	36 052 (18.0)	74 493 (17.9)	58 425 (17.6)	8217 (19.5)	1346 (19.5)
ADHD^e^	3590 (1.8)	7101 (1.7)	5316 (1.6)	776 (1.8)	143 (2.1)
PTSD^e^	3126 (1.6)	6130 (1.5)	4716 (1.4)	723 (1.7)	134 (1.9)
Bipolar disorder^e^	4218 (2.1)	7683 (1.9)	6055 (1.8)	797 (1.9)	152 (2.2)
Psychosis^e^	2057 (1.0)	3915 (0.9)	2972 (0.9)	492 (1.2)	94 (1.4)
Modified Elixhauser comorbidity score^f^					
0	122 953 (61.5)	245 876 (59.2)	196 573 (59.2)	24 051 (56.9)	3970 (57.7)
1	48 399 (24.2)	105 565 (25.4)	84 842 (25.5)	11 023 (26.1)	1756 (25.5)
2	19 456 (9.7)	43 523 (10.5)	34 998 (10.5)	4708 (11.1)	785 (11.4)
≥3	9027 (4.5)	20 159 (4.9)	15 708 (4.7)	2464 (5.8)	375 (5.4)
Years					
2010-2015	130 843 (65.5)	241 320 (58.1)	192 958 (58.1)	23 336 (55.2)	3851 (55.9)
2016-2018	68 992 (34.5)	173 803 (41.9)	139 163 (41.9)	18 910 (44.8)	3035 (44.1)
Census region					
Northeast	15 551 (7.8)	32 570 (7.8)	26 248 (7.9)	3183 (7.5)	586 (8.5)
Midwest	38 201 (19.1)	80 536 (19.4)	64 092 (19.3)	8103 (19.2)	1272 (18.5)
South	98 605 (49.3)	201 315 (48.5)	160 026 (48.2)	20 391 (48.3)	3276 (47.6)
West	46 313 (23.2)	98 616 (23.8)	80 088 (24.1)	10 328 (24.4)	1641 (23.8)
Unknown	1165 (0.6)	2086 (0.5)	1667 (0.5)	241 (0.6)	111 (1.6)
Insurance plan type					
Commercial	75 392 (37.7)	144 528 (34.8)	108 558 (32.7)	14 009 (33.2)	2181 (31.7)
Medicare Advantage	124 443 (62.3)	270 595 (65.2)	223 563 (67.3)	28 237 (66.8)	4705 (68.3)

Abbreviations: ADHD, attention-deficit/hyperactivity disorder; MME, morphine milligram equivalent; PTSD, posttraumatic stress disorder.

^a^ For this comparison, month 4 was used, which was the first month after the treatment assignment period where noncensored episodes were assigned a mutually exclusive treatment strategy (stable dosage, taper, or discontinued).

^b^ Individuals could contribute multiple episodes if they met the criteria for a stable long-term opioid episode in more than 1 year. For the Patients column, we analyzed the first qualifying episode for individuals with multiple episodes.

^c^ Baseline MME represents the mean daily MME in the baseline month, the final month of the 6-month eligibility assessment period.

^d^ Presence of 1 or more claims for a benzodiazepine or gabapentinoid (gabapentin or pregabalin) prescription in the 6-month eligibility assessment period.

^e^ Presence of 1 or more claims with a diagnosis code for listed comorbidity in the 6-month eligibility assessment period.

^f^ Elixhauser comorbidity score modified to exclude mental health and substance use diagnostic categories that were included as separate covariates.

After the treatment assignment period, 87.1% of episodes were considered stable, 11.1% were considered a taper, and 1.8% were considered abrupt discontinuation. Baseline characteristics were relatively balanced among treatment strategies, with some exceptions. A higher proportion of episodes in the abrupt discontinuation group were among individuals 18 to 44 years of age (16.0%) compared with 13.2% for taper and 12.4% for stable dosage. Of those with abrupt discontinuation, more had a baseline MME of 50 to 89 mg (43.2%) compared with 37.7% for stable dosage and 32.4% for taper, while fewer individuals with abrupt discontinuation had baseline MME of 200 mg or more (20.5%) compared with 25.5% for stable dosage and 29.6% for taper.

### Intention-to-Treat Effect

**Figure 2.**
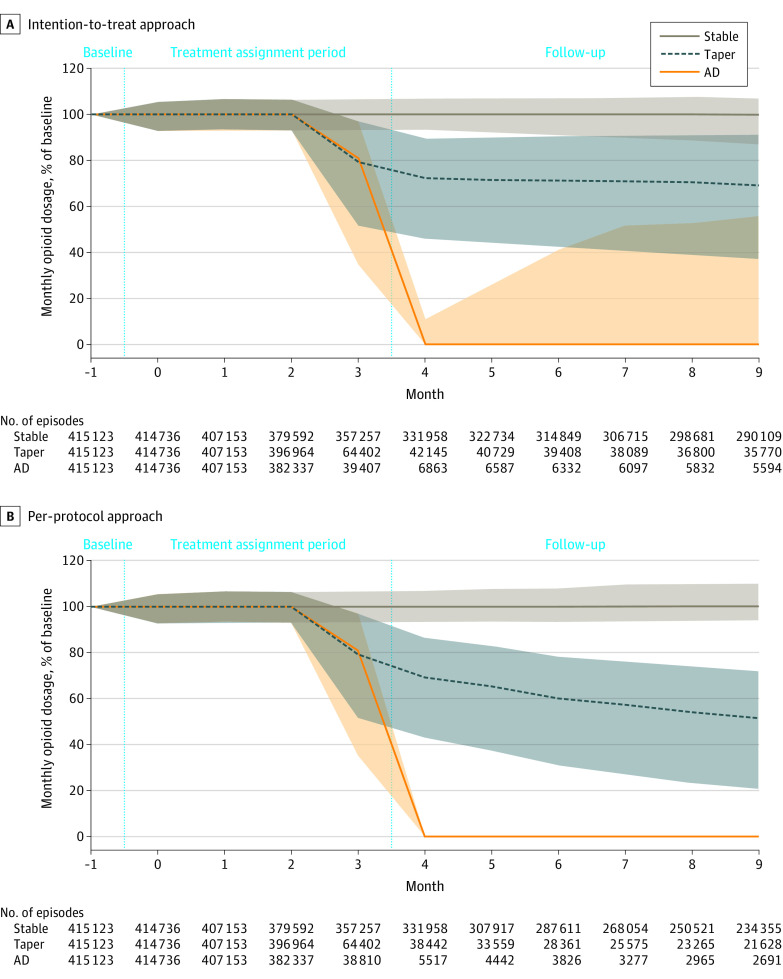
Unadjusted Dosage Trajectories Presented as Percentage of Baseline Dosage by Treatment Strategy During the Treatment Assignment Period and Follow-up The lines and shaded areas indicate median and IQR. AD indicates abrupt discontinuation. The vertical dotted lines separate the time periods into baseline, treatment assignment, and follow-up periods.

For the stable dosage group, the unweighted observed median opioid dosage was 100% of baseline in each follow-up month (Figure 2A). For the taper group, the median dosage was 72.3% of baseline in month 4, decreasing to 69.2% of baseline in month 9. The median dosage of the abrupt discontinuation group was 0 MME in each follow-up month; however, some patients resumed taking opioids, with the 75th percentile dosage reaching 55.8% of baseline by month 9.

We observed 5835 opioid overdose or suicide events during 7 466 287 person-months of follow-up for an unadjusted incidence of 0.94 events per person-year. The adjusted cumulative incidence of opioid overdose or suicide 11 months after baseline associated with a stable dosage strategy was 0.96% (95% CI, 0.92%-0.99%), with a taper strategy was 1.10% (95% CI, 0.99%-1.22%), and with an abrupt discontinuation strategy was 1.28% (95% CI, 0.93%-1.38%) (Table 2). The risk difference associated with taper vs stable dosage was 0.15% (95% CI, 0.03%-0.26%) and with abrupt discontinuation vs stable dosage was 0.33% (95% CI, −0.03% to 0.74%). The risk ratio associated with taper vs stable dosage was 1.15 (95% CI, 1.04-1.27) and with abrupt discontinuation vs stable dosage was 1.34 (95% CI, 0.97-1.79).

**Table 2.** Adjusted Cumulative Incidence at Month 10 for Each Treatment Strategy and Absolute Risk Differences and Risk Ratios for Primary, Secondary, and Stratified Analyses

Outcome	% (95% CI)					Risk ratio [vs stable dosage] (95% CI)	
	Adjusted cumulative incidence			Absolute risk difference (vs stable dosage)		Taper	Abrupt discontinuation
	Stable dosage	Taper	Abrupt discontinuation	Taper	Abrupt discontinuation		
Primary composite outcome (opioid overdose or suicide)							
Intention-to-treat approach	0.96 (0.92 to 0.99)	1.10 (0.99 to 1.22)	1.28 (0.93 to 1.38)	0.15 (0.03 to 0.26)	0.33 (−0.03 to 0.74)	1.15 (1.04 to 1.27)	1.34 (0.97 to 1.79)
Per-protocol approach	0.93 (0.89 to 0.96)	1.15 (1.02 to 1.29)	1.03 (0.64 to 1.47)	0.22 (0.10 to 0.36)	0.10 (−0.29 to 0.54)	1.24 (1.11 to 1.39)	1.11 (0.69 to 1.58)
Secondary outcomes (intention-to-treat approach)							
Opioid overdose	0.35 (0.33 to 0.37)	0.41 (0.34 to 0.48)	0.44 (0.23 to 0.69)	0.06 (−0.01 to 0.13)	0.09 (−0.11 to 0.35)	1.16 (0.99 to 1.37)	1.26 (0.68 to 2.03)
Suicide	0.63 (0.60 to 0.66)	0.73 (0.65 to 0.83)	0.98 (0.62 to 1.36)	0.10 (0.01 to 0.20)	0.35 (−0.01 to 0.72)	1.16 (1.01 to 1.30)	1.55 (0.99 to 2.13)
Stratified analyses (intention-to-treat approach, primary combined outcome)							
Baseline MME, mg							
50-89	0.69 (0.64 to 0.73)	0.62 (0.48 to 0.79)	0.79 (0.34 to 1.42)	−0.07(−0.20 to 0.10)	0.10 (−0.36 to 0.76)	0.89 (0.79 to 1.14)	1.14 (0.48 to 2.09)
90-199	1.02 (0.96 to 1.08)	1.23 (1.04 to 1.42)	1.65 (0.99 to 2.55)	0.21 (0.02 to 0.40)	0.63 (−0.04 to 1.52)	1.20 (1.02 to 1.40)	1.62 (0.96 to 2.49)
≥200	1.25 (1.16 to 1.33)	1.64 (1.49 to 1.88)	1.33 (0.65 to 2.14)	0.39 (0.15 to 0.64)	0.08 (−0.60 to 0.91)	1.31 (1.12 to 1.52)	1.07 (0.53 to 1.74)
Baseline year							
2010-2015	0.83 (0.79 to 0.87)	1.01 (0.87 to 1.16)	1.28 (0.76 to 1.87)	0.18 (0.04 to 0.33)	0.45 (−0.08 to 1.04)	1.22 (1.05 to 1.41)	1.54 (0.90 to 2.25)
2016-2018	1.12 (1.06 to 1.19)	1.24 (1.07 to 1.42)	1.20 (0.69 to 1.78)	0.12 (−0.05 to 0.30)	0.07 (−0.42 to 0.67)	1.10 (0.96 to 1.27)	1.06 (0.64 to 1.59)

Abbreviation: MME, morphine milligram equivalent.

**Figure 3.**
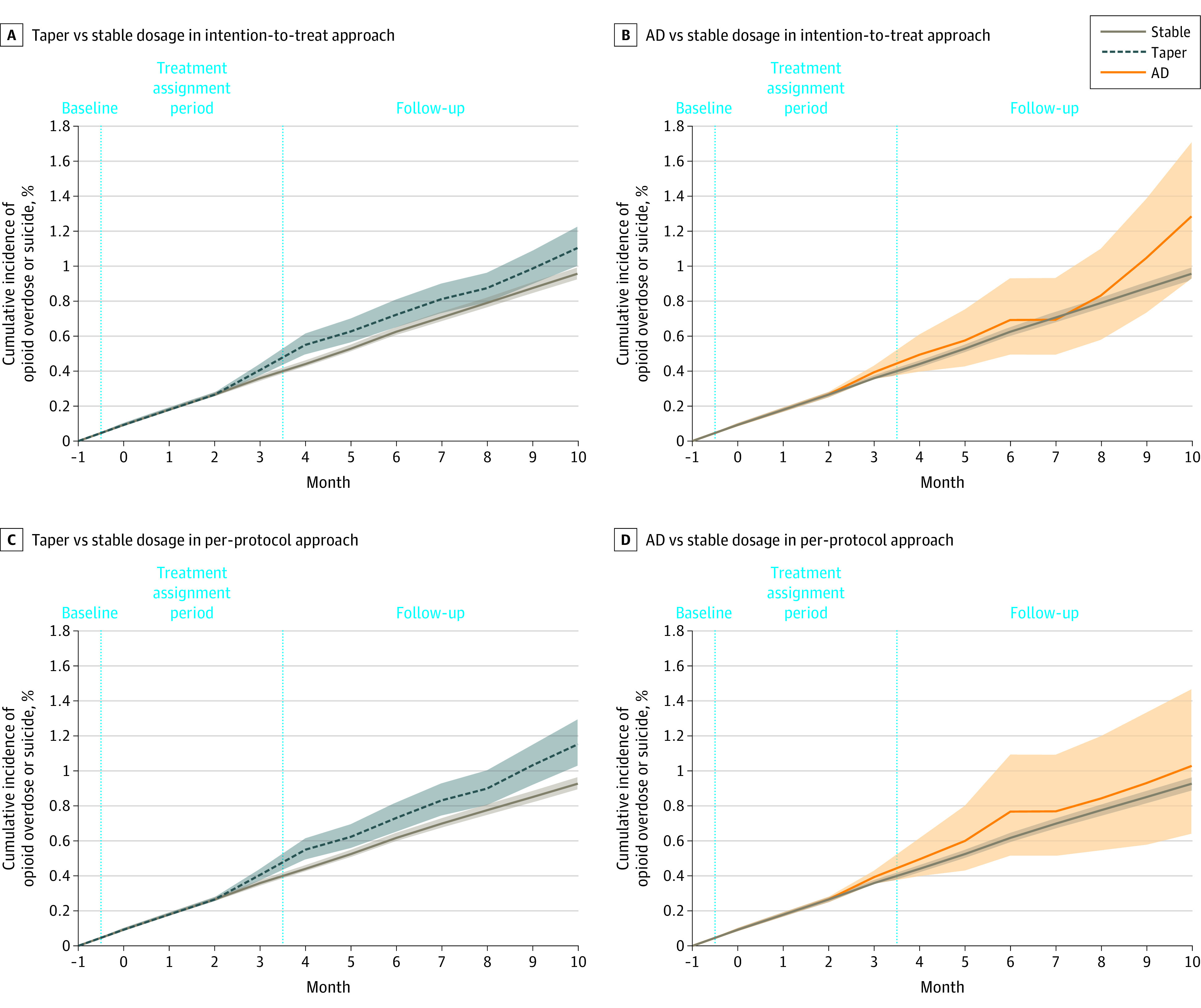
Adjusted Cumulative Incidence of Opioid Overdose or Suicide Event by Treatment Strategy AD indicates abrupt discontinuation. The shaded areas indicate 95% CI. The vertical dotted lines separate the time periods into baseline, treatment assignment, and follow-up periods.

Adjusted cumulative incidence curves for overdose or suicide diverged for the comparison of stable dosage and taper at month 4, with a higher incidence associated with the taper vs stable dosage treatment strategies thereafter (Figure 3A). For the comparison of stable dosage vs abrupt discontinuation treatment strategies, the 95% CIs overlapped throughout the follow-up period (Figure 3B), indicating similar event rates.

### Per-Protocol Effect

With the per-protocol approach, the opioid dosage remained stable in the stable dosage group and was 0 mg MME throughout follow-up in the abrupt discontinuation group (Figure 2B). In the taper group, the median dosage continued to decrease throughout follow-up from 69.2% of baseline at month 4 to 51.5% of baseline at month 9 of follow-up. The adjusted cumulative incidence curves and absolute risk differences for the primary outcomes associated with comparisons of taper and stable dosage and abrupt discontinuation and stable dosage strategies were similar to the intention-to-treat approach (Figure 3C and D; Table 2).

### Secondary Outcomes and Stratified and Sensitivity Analyses

The cumulative incidences of opioid overdose and suicide associated with the stable dosage strategy were 0.35% (95% CI, 0.33%-0.37%) and 0.63% (95% CI, 0.60%-0.66%), respectively, at 11 months (Table 2). Compared with stable dosage, tapering was associated with a small increase in risk of suicide (0.10%; 95% CI, 0.01%-0.20%), but no other treatment strategies yielded significant associations for secondary outcomes of opioid overdose or suicide.

In the stratified analyses, the absolute risk difference for the combined outcome of suicide or overdose associated with the taper vs stable dosage groups increased with increasing baseline MME: −0.07% (95% CI, −0.20% to 0.10%) for 50 to 89 mg per day, 0.21% (95% CI, 0.02%-0.40%) for 90 to 199 mg per day, and 0.39% (95% CI, 0.15%-0.64%) for 200 mg or more per day (Table 2). Associated risk differences between treatment groups were similar when comparing treatment episodes from 2010 to 2015 with those from 2016 to 2018. Sensitivity analyses that trimmed weights and used an alternative treatment assignment scheme produced similar results overall (eTable 5 in the Supplement).

## Discussion

In this emulated trial including more than 400 000 episodes of stable long-term opioid therapy, opioid tapering was associated with a small (0.15%) absolute increase in the risk of overdose or suicide events compared with a stable dosage during 11 months of follow-up. We did not identify a difference in the risk of overdose or suicide events between abrupt discontinuation and stable dosage, although the smaller number of episodes categorized as abrupt discontinuation may have reduced precision. The findings were robust to secondary and sensitivity analyses.

The risk ratio of 1.15 for opioid overdose or suicide events associated with opioid tapering was smaller than in past studies conducted in other populations. This study examined commercially insured individuals receiving a stable long-term opioid dosage without evidence of opioid misuse. A large study of Veterans Health Administration patients estimated adjusted hazard ratios between 1.7 and 6.8 for the association of treatment discontinuation with suicide or overdose among patient subgroups defined by length of prior treatment.^17^ A study of Oregon Medicaid recipients found adjusted hazard ratios for suicide of 3.6 for discontinuation and 4.5 for tapering.^18^ A study using the same claims data set and similar definition of long-term opioid therapy as our study identified effect estimates between those in our study and those in prior studies, with an estimated adjusted incidence rate ratio of 1.3 for the association of dose tapering with overdose and 2.4 for the association of dose tapering with suicide attempts.^32^

The present study offers 2 key methodological advances in the study of opioid tapering. First, we aligned treatment assignment and the start of follow-up through the use of clones.^22,33^ Without this process, follow-up must be started before treatment assignment, introducing immortal time bias as individuals must survive long enough to receive treatment, or follow-up must be started after treatment is identified, resulting in a survival bias. Second, we excluded patients for whom tapering may not be clinically appropriate; for example, we excluded patients with evidence for opioid misuse who should have additional intervention, including evaluation for and treatment of OUD.^4^ Overall, the much smaller associations observed in the present study suggest that the high estimates of harm from opioid tapering and discontinuation relative to stable dosages found in prior studies may have, at least in part, been the result of residual confounding and biases.

Although these data show a more neutral association of opioid tapering with opioid overdose or suicide compared with past studies, they do not show a protective association. Overall, evidence does not suggest that tapering opioid dosages achieves the goal of reducing the risk of harms associated with long-term opioid therapy. Policies establishing dosage thresholds or mandating tapers for all patients receiving long-term opioid therapy are not supported by existing data in terms of anticipated benefits even if, as we found, the rate of adverse outcomes is small. Health systems and clinicians must continue to develop and implement patient-centered approaches to pain management for patients with established long-term opioid therapy.^34^ Clinically recommended approaches include discussing patients’ experiences with both positive and negative associations of long-term opioid therapy and other pain management strategies, developing risk reduction plans and addiction-informed treatment strategies for patients with signs of OUD, and facilitating voluntary opioid tapering for patients who are interested in lowering their dosages.^35,36,37^

### Limitations

This study has some limitations. First, our cohort was limited to commercially insured individuals without signs of opioid misuse prior to tapering, and the results may not be generalizable to other settings and populations. Second, we did not have information on the clinical intent or motivation of tapering, and opioid tapering was inferred from observed dosage trajectories. Third, as in any observational study, the results are subject to unmeasured confounding. We strove to address this factor in several ways through a clear delineation of inclusion or exclusion criteria to focus on patients receiving long-term opioid therapy without evidence of high-risk use behaviors in the baseline period. Fourth, we could observe only overdose or suicide outcomes that were captured in medical claims data, and events that led to death without a medical encounter were not captured.

## Conclusions

We emulated a target trial of opioid tapering and abrupt discontinuation among a large cohort of commercially insured individuals receiving stable long-term opioid therapy without indicators of problem opioid use. Although the estimates of harm associated with opioid tapering were much smaller in this study than prior observational studies, our findings do not indicate that policies of mandatory opioid dosage tapering will reduce short-term harm via suicide and overdose. More clinically informed and patient-centered strategies are needed to reduce harms associated with long-term opioid therapy.

## Notes

Supplement.
eAppendix.
**eTable 1.** Diagnosis Codes to Identify Study Outcomes and Exclusion Criteria **eTable 2.** Codes to Identify Drug Detoxification, MOUD Administration, and Hospice **eTable 3.** List of Medications Included as Benzodiazepines and Gabapentinoids Identified via NDC Codes Using the MicroMedex REDBook, Accessed on 2/16/2021 **eTable 4.** Diagnosis Codes to Identify Behavioral Health Comorbidities **eTable 5.** Cumulative Incidence, Absolute Risk Difference, and Risk Ratio for Primary Outcomes for Main Approach and Sensitivity Analyses **eFigure.** Illustrative Examples of Treatment Assignment, Cloning Procedures, and Censoring
eReferences.
