# Supplementary material for: Comparative Effectiveness of Opioid Tapering or Abrupt Discontinuation vs No Dosage Change for Opioid Overdose or Suicide for Patients Receiving Stable Long-term Opioid Therapy
Source: JAMA Netw Open. 2022 Aug 12;5(8):e2226523. doi: 10.1001/jamanetworkopen.2022.26523 (PMC9375167; doi:10.1001/jamanetworkopen.2022.26523)
Supplement: Supplement. — eAppendix. eTable 1. Diagnosis Codes to Identify Study Outcomes and Exclusion Criteria eTable 2. Codes to Identify Drug Detoxification, MOUD Administration, and Hospice eTable 3. List of Medications Included as Benzodiazepines and Gabapentinoids Identified via NDC Codes Using the MicroMedex REDBook, Accessed on 2/16/2021 eTable 4. Diagnosis Codes to Identify Behavioral Health Comorbidities eTable 5. Cumulative Incidence, Absolute Risk Difference, and Risk Ratio for Primary Outcomes for Main Approach and Sensitivity Analyses eFigure. Illustrative Examples of Treatment Assignment, Cloning Procedures, and Censoring eReferences. [file jamanetwopen-e2226523-s001.pdf]

Supplementary Online Content

Larochelle MR, Lodi S, Yan S, Clothier BA, Goldsmith ES, Bohnert ASB. Comparative effectiveness of opioid tapering or abrupt discontinuation vs no dosage change for opioid overdose or suicide for patients receiving stable long-term opioid therapy. *JAMA Netw Open.* 2022;5(8):e2226523. doi:10.1001/jamanetworkopen.2022.26523

eAppendix.
eTable 1. Diagnosis Codes to Identify Study Outcomes and Exclusion Criteria
eTable 2. Codes to Identify Drug Detoxification, MOUD Administration, and Hospice
eTable 3. List of Medications Included as Benzodiazepines and Gabapentinoids Identified via NDC Codes Using the MicroMedex REDBook, Accessed on 2/16/2021
eTable 4. Diagnosis Codes to Identify Behavioral Health Comorbidities
eTable 5. Cumulative Incidence, Absolute Risk Difference, and Risk Ratio for Primary Outcomes for Main Approach and Sensitivity Analyses
eFigure. Illustrative Examples of Treatment Assignment, Cloning Procedures, and Censoring
eReferences.

This supplementary material has been provided by the authors to give readers additional information about their work.

© 2022 Larochelle MR et al. *JAMA Network Open.*

eAppendix.

Calculating daily Morphine Milligram Equivalents (MME).

We used the CDC Opioid NDC and Oral MME Conversion File to identify opioid analgesics by National Drug Code (NDC) and their associated MME conversion factors.[1] We excluded liquid antitussive and intravenous formulations not used for outpatient treatment of pain, and buprenorphine given lack of an agreed upon conversion factor. Pharmacy claims contain the quantity dispensed and days supply for each prescription. We calculated the MME for each prescription by multiplying the quantity by the strength and MME conversion factor. We attributed the MME equally over the days supply starting with the dispensing date. We summed the MME on days where more than one prescription overlapped. We calculated the mean MME for each calendar month by dividing the sum of the MME on each day divided by the number of days in each month.

Defining early refills.

We defined an early refill as a medication for the same opioid formulation and strength (e.g. 5 mg oxycodone tablets) filled more than 3 days prior to the run out date (dispensing date plus days supply) of the prior prescription.

Specification of regression models to derive weights.

Treatment weights were calculated to adjust for informative censoring of clones during the treatment assignment period. We developed separate logistic regression models for each month and treatment strategy, controlling for potential confounders identified in the Methods, and used them to calculate the predicted probability of complying with the assigned strategy in

© 2022 Larochelle MR et al. *JAMA Network Open.*

each month of the treatment assignment period. The treatment weights were calculated as the inverse of the predicted probability of complying with the assigned strategy.

Censoring weights were derived to adjust for selection bias from loss to follow-up. We used a pooled logistic regression model to estimate the probability of not being disenrolled from the health plan adjusting for potential confounders plus a term for month. Censoring weights were defined as the inverse of the probability of being uncensored in each month.

Treatment adherence weights were used only the per protocol analysis to adjust for confounders of lack of treatment adherence. We used a pooled logistic regression model to estimate the probability of not being censored due to lack of adherence to the assigned treatment strategy adjusting for potential confounders plus a term for month. Treatment adherence weights were calculated as the inverse probability of remaining adherent to the assigned treatment in each month.

© 2022 Larochelle MR et al. *JAMA Network Open.*

eTable 1. Diagnosis Codes to Identify Study Outcomes and Exclusion Criteria

**Opioid Overdose**	
ICD-9	965.0*, E850.0, E850.1, E850.2
ICD-10	T40.0X1, T40.0X2, T40.0X4, T40.0X5, T40.1X1, T40.1X2, T40.1X4, T40.2X1, T40.2X2, T40.2X4, T40.2X5, T40.3X1, T40.3X2, T40.3X4, T40.3X5, T40.4X1, T40.4X2, T40.4X4, T40.4X5
**Suicide**	
ICD-9	E950-E959
ICD-10	X71-X83,<br><br>T14.91<br><br>[The following codes require a 6th character of "2" to specify intentional events]: T36.0-T36.8, T37.0-T39.8, T40.0-T41.3, T41.5-T42.6, T42.8-T43.8, T44.0-T45.8, T46.0-T47.8, T48.0-T49.8 T51.0-T51.8, T52.0-T52.8, T53.0-T53.8, T54.0-T54.8, T55.0-T56.8, T57.0-T57.8, T58.2-T58.8, T59.0-T59.8, T60.0-T60.8, T61.2-T61.8, T62.0-T62.8, T63.0-T63.8, T64.1-T64.7, T64.9-T65.8, T71<br><br>[The following codes require a 5th character of "2" to specify intentional events]: T36.9, T37.9, T39.9, T41.4, T42.7, T43.9, T45.9, T47.9, T49.9, T51.9, T52.9, T53.9, T54.9, T56.9, T57.9, T58.0, T58.1, T58.9, T59.9, T60.9, T61.0, T61.1, T61.9, T62.9, T63.9, T64.0, T64.8, T65.9
**Hepatitis C**	
ICD-9	070.41, 070.44, 070.51, 070.54, 070.70, 070.71, V02.62
ICD-10	B17.10, B17.11, B18.2, B19.20, B19.21, Z22.52
**Cancer except Non-melanoma Skin Cancer**	
ICD-9	140.x-208.x(except 173.x), 209.0-209.3, V10.x (except V10.83)
ICD-10	C00.x-C80.x (except C44.x, include C7A.x, exclude C7B.x)
**Non-tobacco Substance Use Disorder**	
ICD-9	303.x, 304.x, 305.0x, 305.2x-305.9x
ICD-10	F10.1x-F10.2x, F11.1x-F11.2x, F12.1x-F12.2x, F13.1x-F13.2x, F14.1x-F14.2x, F15.1x-F15.2x, F16.1x-F16.2x, F18.1x-F18.2x, F19.1x-F19.2x
**Infectious Endocarditis or Septic Arthritis (Serious Injection-Related Infections)**	
ICD-9	112.81, 421.0, 421.1, 421.9, 424.9*, 711.0*
ICD-10	B37.6, I33.0, I33.9, I38, I39, M00.0*, M00.2*, M00.8*, M00.9

* - indicates inclusion of all codes under parent code

© 2022 Larochelle MR et al. *JAMA Network Open.*

eTable 2. Codes to Identify Drug Detoxification, MOUD Administration, and Hospice

**Drug Detoxification**	
Revenue	116, 126, 136, 146, 156, 1002
ICD-10	HZ2ZZZZ
HCPCS	H0009, H0010, H0011, H0012
**MOUD Administration**	
HCPCS	H0020, J0570-J0575, J0592, J2315
**MOUD Pharmacy Claim**	
NDC	NDC code for buprenorphine or buprenorphine/naloxone indicated for OUD identified via the CDC Opioid NDC and Oral MME Conversion File[1] or naltrexone identified via Micromedex RED BOOK[2] respectively
**Hospice**	
Revenue	115, 0125, 0135, 0145, 0155, 0235, 0650, 0651, 0652, 0653, 0654, 0655, 0656, 0657, 0658, 0659
HCPCS	99377, 99378, G0182, G0337, G9474, G9475, G9476, G9477, G9478, G9479, G9524, Q5001, Q5002, Q5003, Q5004, Q5005, Q5006, Q5007, Q5008, Q5009, Q5010, S0255, S9126, T2042, T2043, T2044, T2045, T2046

© 2022 Larochelle MR et al. *JAMA Network Open.*

eTable 3. List of Medications Included as Benzodiazepines and Gabapentinoids Identified via NDC Codes Using the MicroMedex REDBook, Accessed on 2/16/2021.[2]

Benzodiazepines	diazepam, clorazepate, oxazepam, lorazepam, alprazolam, clonazepam, midazolam, triazolam, estazolam, temazepam, chlordiazepoxide, flurazepam, clobazam, quazepam
Gabapentinoids	Gabapentin, gabapentin enacarbil, pregabalin

© 2022 Larochelle MR et al. *JAMA Network Open.*

eTable 4. Diagnosis Codes to Identify Behavioral Health Comorbidities

**Depression**	
ICD-9	296.2, 296.20-296.26, 296.3, 296.30-296.36, 300.4, 311
ICD-10	F32, F32.0-F32.5, F32.9, F33, F33.0-F33.3, F33.40-F33.42, F33.9, F34.1
**Anxiety**	
ICD-9	293.84, 300 ,300.0, 300.00, 300.01, 300.02, 300.04, 300.09, 300.2, 300.20, 300.21, 300.22, 300.23, 300.29, 309.21, 313, 313.0, 313.00, 313.2, 313.21, 313.22, 313.23, 313.8 ,313.82, 313.9
ICD-10	F06.4, F40.00, F40.01, F40.02, F40.1, F40.10, F40.11, F40.2, F40.210, F40.218, F40.220, F40.228, F40.23, F40.230, F40.231, F40.232, F40.233, F40.24, F40.240, F40.241, F40.242, F40.243, F40.248, F40.29, F40.290, F40.298, F40.8, F40.9, F41, F41.0, F41.1, F41.3, F41.8, F41.9, F93.0, F94.0
**ADHD**	
ICD-9	314.00, 314.01
ICD-10	F90, F90.0, F90.1 ,F90.2, F90.8, F90.9
**PTSD**	
ICD-9	308, 308.0, 308.1, 308.2, 308.3, 308.30, 308.9, 309.81, 309.89
ICD-10	F43.0, F43.10, F43.11, F43.12, F43.8, F43.9, F94.1, F94.2
**Bipolar**	
ICD-9	296, 296.0, 296.00-296.06, 296.1, 296.10-296.16, 296.4, 296.40-296.46, 296.5, 296.50-296.56, 296.6, 296.60-296.66, 296.7, 296.70, 296.75, 296.8, 296.80-296.83, 296.89, 301.13
ICD-10	F30.10-F30.13, F30.2-F30.4, F30.8, F30.9, F31*, F34.0
**Psychosis**	
ICD-9	295.3, 295.30-295.35, 293.81, 293.82, 293.89, 295, 295.0, 295.10-295.15, 295.2, 295.20-295.25, 295.40-295.45, 295.50-295.55, 295.6, 295.60-295.65, 295.7, 295.70-295.75, 295.80-295.85, 295.9, 295.90-295.95, 297.0, 297.1, 297.10, 297.2, 297.3, 297.8, 297.9, 298.0, 298.1, 298.2, 298.3, 298.4, 298.8, 298.80, 298.9, 298.90, 293.81, 293.82, 293.89, 295, 295.0, 295.10-295.15, 295.2, 295.20-295.25, 295.3, 295.30-295.35, 295.40-295.45, 295.50-295.55, 295.6, 295.60-295.65, 295.7, 295.70-295.75, 295.80-295.85, 295.9, 295.90-295.95, 297.0, 297.1, 297.10, 297.2, 297.3, 297.8, 297.9, 298.0-298.4, 298.8, 298.80, 298.9, 298.90
ICD-10	F06.0, F06.1, F06.2, F06.3, F20.0, F20.1, F20.2, F20.3, F20.5, F20.81, F20.89, F20.9, F22, F23, F24, F25, F25.0, F25.1, F25.8, F25.9, F28, F29, F53, F06.0, F06.1, F06.2, F06.3, F20.0, F20.1, F20.2, F20.3, F20.5, F20.81, F20.89, F20.9, F22, F23, F24, F25, F25.0, F25.1, F25.8, F25.9, F28, F29, F53

* - indicates inclusion of all codes under parent code

© 2022 Larochelle MR et al. *JAMA Network Open.*

eTable 5. Cumulative Incidence, Absolute Risk Difference, and Risk Ratio for Primary Outcomes for Main Approach and Sensitivity Analyses

	Cumulative Incidence (at month 10)			Absolute Risk Difference (vs Stable)		Risk Ratio (vs Stable)	
	Stable	Taper	Abrupt Discontinuation	Taper	Abrupt Discontinuation	Taper	Abrupt Discontinuation
Primary Composite Outcome (opioid overdose or suicide)							
Intent-to-treat	0.96% (0.92%,0.99%)	1.10% (0.99%,1.22%)	1.28% (0.93%,1.38%)	0.15% (0.03%,0.26%)	0.33% (-0.03%,0.74%)	1.15 (1.04,1.27)	1.34 (0.97,1.79)
Per protocol	0.93% (0.89%,0.96%)	1.15% (1.02%,1.29%)	1.03% (0.64%,1.47%)	0.22% (0.10%,0.36%)	0.10% (-0.29%,0.54%)	1.24 (1.11,1.39)	1.11 (0.69, 1.58)
Sensitivity analyses (intent-to-treat approach, primary combined outcome)							
Trimmed weights[a]	0.96% (0.92%, 0.99%)	1.11% (1.01%, 1.24%)	1.12% (0.87%, 1.45%)	0.16% (0.05%, 0.27%)	0.16% (-.01%, 0.49%)	1.17 (1.05,1.29)	1.18 (0.90,1.51)
Tx assignment[b]	0.88% (0.82%, 0.89%)	0.89% (0.86%, 0.92%)	0.85% (0.82%, 0.89%)	0.04% (0.01%, 0.06%)	0.00% (0.00%, 0.01%)	1.04 (1.02,1.07)	1.00 (0.99,1.01)

[a] In this sensitivity analysis, weights were trimmed at 99% to reduce potential impact of outliers.

[b] In this sensitivity analysis, tapering was defined as a single month (rather than two consecutive months) of a dose decrease of 15% more than baseline during the treatment assignment period, and abrupt discontinuation was defined as a month meeting tapering criteria with a mean MMME of 0 mg per day.

© 2022 Larochelle MR et al. *JAMA Network Open.*

eFigure. Illustrative Examples of Treatment Assignment, Cloning Procedures, and Censoring

Legend:

100% Mean dose as % of baseline month; >85%; 1-85%; 0%

A value of 1 indicates clone remains in the analysis, and 0 indicates censoring.

Censoring in per protocol approach when dose no longer consistent with treatment strategy.

	Baseline Period	Treatment Assignment Period				Follow-up Period					
Month:	-1	0	1	2	3	4	5	6	7	8	9

Example 1: Stable.

	-1	0	1	2	3	4	5	6	7	8	9
Dose	100%	100%	90%	95%	105%	95%	105%	95%	100%	95%	110%
CLONES: Stable		1	1	1	1	1	1	1	1	1	1
CLONES: Taper		1	1	0	0	0	0	0	0	0	0
CLONES: Abrupt Discontinuation		1	1	0	0	0	0	0	0	0	0

Example 2: Abrupt Discontinuation

	-1	0	1	2	3	4	5	6	7	8	9
Dose	100%	100%	80%	0%	0%	0%	0%	0%	0%	0%	0%
CLONES: Stable		1	1	0	0	0	0	0	0	0	0
CLONES: Taper		1	1	0	0	0	0	0	0	0	0
CLONES: Abrupt Discontinuation		1	1	1	1	1	1	1	1	1	1

Example 3: Taper

	-1	0	1	2	3	4	5	6	7	8	9
Dose	100%	84%	70%	65%	60%	50%	50%	50%	50%	50%	50%
CLONES: Stable		1	0	0	0	0	0	0	0	0	0
CLONES: Taper		1	1	1	1	1	1	1	1	1	1
CLONES: Abrupt Discontinuation		1	0	0	0	0	0	0	0	0	0

Example 4: Stable

	-1	0	1	2	3	4	5	6	7	8	9
Dose	100%	100%	90%	80%	105%	95%	105%	95%	70%	65%	60%
CLONES: Stable		1	1	1	1	1	1	1	1	1	1
CLONES: Taper		1	1	1	0	0	0	0	0	0	0
CLONES: Abrupt Discontinuation		1	1	1	0	0	0	0	0	0	0

Example 5: Taper

	-1	0	1	2	3	4	5	6	7	8	9
Dose	100%	100%	0%	80%	105%	95%	105%	95%	100%	95%	110%
CLONES: Stable		1	1	0	0	0	0	0	0	0	0
CLONES: Taper		1	1	1	1	1	1	1	1	1	1
CLONES: Abrupt Discontinuation		1	1	0	0	0	0	0	0	0	0

Example 6: Abrupt Discontinuation

	-1	0	1	2	3	4	5	6	7	8	9
Dose	100%	100%	80%	0%	0%	0%	60%	95%	100%	95%	110%
CLONES: Stable		1	1	0	0	0	0	0	0	0	0
CLONES: Taper		1	1	0	0	0	0	0	0	0	0
CLONES: Abrupt Discontinuation		1	1	1	1	1	1	1	1	1	1

© 2022 Larochelle MR et al. *JAMA Network Open.*

eReferences.

1. National Center for Injury Prevention and Control. CDC compilation of benzodiazepines, muscle relaxants, stimulants, zolpidem, and opioid analgesics with oral morphine milligram equivalent conversion factors, 2019 version. 2019; Available at https://www.cdc.gov/drugoverdose/resources/data.html.
2. IBM Watson Health. IBM Micromedex Red Book [Internet]. 2021.

© 2022 Larochelle MR et al. *JAMA Network Open.*
